# Highlight: Extreme Genetic Drift in the Maniq People of Southern Thailand

**DOI:** 10.1093/gbe/evac041

**Published:** 2022-04-13

**Authors:** Casey McGrath

Residing in the hills of southern Thailand, the Maniq comprise one of the last hunter–gatherer communities in the world. Although the Maniq are geographically isolated, they share many cultural features with the Semang peoples, most of whom live over the border in Malaysia. Due to the complex relationships among the various communities in mainland Southeast Asia, anthropologists have long debated the demographic history of the area, with one, two, three, or four waves of human migration having been proposed for the region. A recent study in *Genome Biology and Evolution* by Göllner et al. (2022) titled “Unveiling the genetic history of the Maniq, a primary hunter-gatherer society” provides new insight into the Maniq and their relationships with other indigenous groups in mainland Southeast Asia.

The international team of researchers from the University of Vienna in Austria, the Uppsala University in Sweden, and the Khon Kaen University in Thailand studied 2.3 million single-nucleotide polymorphisms in 11 Maniq individuals who agreed to participate in the study. While a relatively small sample, this represents over 3% of the current Maniq population of ∼250 individuals. The team then compared the data from the Maniq with both present-day populations and ancient DNA samples collected in the region.

**Fig. 1.**
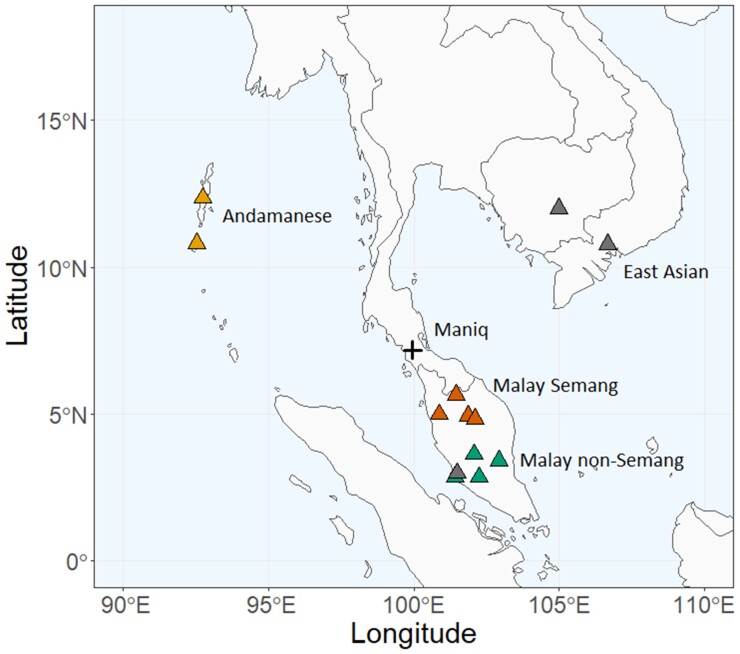
Map with approximate locations of the Maniq and other nearby populations included in the study.

“One of our main conclusions is that the Maniq are a very secluded community and have been separated from the other Semang for quite some time,” says Göllner, first author of the study. As suggested by their cultural ties, the Maniq appeared to be most closely related to the Semang groups in Malaysia, indicating a recent shared history (fig. 1). Comparisons with other modern groups showed that the Maniq and Malay Semang populations shared alleles with indigenous Papuans and Andamanese, indicating “deep historical relationships among these populations,” according to the study’s authors.

The study also revealed that the Maniq exhibited similarities to ancient DNA samples associated with the Hòabìnhian, a cultural complex of ancient hunter–gatherers thought to be the ancestors of present-day hunter–gatherers in mainland Southeast Asia. In the past, Hòabìnhian-related populations were more widespread, with descendants found in Laos, southern China, and even as far as Japan. According to the study’s authors, however, “due to the recent expansion of East Asian-related groups, the Hòabìnhian-related cultural communities were either displaced, replaced, or absorbed into the larger population of farmer migrants.” The study reveals that this was not the case with the Maniq, who remained largely isolated and retained their hunter–gatherer lifestyle, “making them one of the few groups in mainland Asia carrying high levels of Hòabìnhian-related ancestry.”

The study did find evidence for some East Asian ancestry among the Maniq. “The most plausible model for the ancestral source of the Maniq is a combination of both Andamanese and East Asian-related ancestries,” posit the authors, with relative contributions of roughly 65% and 35%, respectively. The researchers estimated that this East Asian ancestry was introduced into the Maniq population approximately 700 years ago, likely via their Malay Semang neighbors.

The most striking finding of the study was the high degree of genetic differentiation exhibited by the Maniq, which was higher than what has been observed for the Mangyan Buhid of the Philippines and comparable to that of the Surui of Brazil, suggesting that the Maniq are more genetically differentiated than virtually any other known human population worldwide. This is likely due to a combination of genetic drift, a long history of geographic and cultural isolation, their historically small population size, and their cultural practice of marrying largely within their own society.

To validate these findings, Göllner hopes to conduct additional studies with larger sample sizes or full genome sequencing data. He notes that the current study and any future work “are only possible thanks to the participation of the Maniq and their long-standing relationship with our cultural anthropologist, Helmut Lukas.” Such studies are made more difficult, however, by the fact that the Maniq currently face several challenges, including intrusion from outsiders, ethnic discrimination, and most notably, the deforestation of the rainforest, limiting their ability to hunt, gather, and follow a traditional lifestyle. Says Göllner, “While solutions will have to be led by the Maniq and other citizens of Thailand, we hope that highlighting the importance of the Maniq will inspire change and help to protect their way of life.”
